# Assessment of fluctuations in wetland ecosystem areas resulting from anthropogenic activities in the Dong Rui commune, Quang Ninh Province, Vietnam

**DOI:** 10.1016/j.heliyon.2023.e16984

**Published:** 2023-06-03

**Authors:** Dung Trung Ngo, Hoi Dang Nguyen, Huan Cao Nguyen

**Affiliations:** a Institute of Tropical Ecology, Joint Vietnam-Russia Tropical Science and Technology Research Center, No. 63, Nguyen Van Huyen Str., Cau Giay District, Hanoi, Viet Nam; b University of Science, Vietnam National University, No. 334, Nguyen Trai Str., Thanh Xuan District, Hanoi, Viet Nam

**Keywords:** Wetland, Variability of cover area, Mangrove forests, Mapping, Policy, Anthropogenic activity

## Abstract

Wetlands are one of the most important ecosystems as habitats for many animal and plant species and are crucial for disaster mitigation, improving environmental quality, storing carbon, and responding to climate change. However, these sensitive ecosystems have been heavily affected by anthropogenic activities, including aquaculture. In this study, we used multitemporal satellite imagery integrated with a verified field survey method to map the coverage of the wetland ecosystem in the Dong Rui commune, Tien Yen district, Quang Ninh Province, Vietnam, five times for four periods from 1975 to 2022, with high accuracy (overall accuracy = 92.3%, Kappa = 0.91). The results showed that from 1975 to 2000, the area of mangrove forests declined sharply (by nearly 2,000 ha), mainly due to policies of development and conversion of land use. From 2000 to 2022, the mangrove forest area was gradually restored, while the area under aquaculture shrank. Anthropogenic impacts, especially the effects of local economic development, and conservation and developmental policies, are the main causes of continuous change in each short period. Our study demonstrates satellite imagery as an effective tool for assessing wetland ecosystem area fluctuations and assessing the extent of human impacts on this natural ecosystem. Our findings can serve as a basis for planning, conservation strategies, and sustainable development of wetland ecosystems and for improving the associated livelihoods of the communities.

## 1 Introduction

Wetlands are defined as areas of shallow water, usually near the soil surface, covered by active plants during the growing season and during periods of water saturation [1]. Wetlands are a broad category that includes coastal wetlands, peatlands, mangroves, estuary wetlands, and marshes [2]. Wetlands and estuaries are biologically highly productive and serve as important habitats for a variety of plants, fish, shellfish, mollusks, and other wildlife. Wetlands also provide flood resistance, protection from storms and wave damage, and improved water quality through agricultural and industrial waste filtration and the refilling of aquifers [3,4]. The importance of wetlands is also reflected in their ability to store carbon and thus mitigate the effects of climate change [5]. Wetlands are estimated to make up approximately 3–6% of the Earth's surface, and those in South America alone account for a large proportion (20% of the continental surface and 42% of the Earth's peatland volume) [6,7]. With an area of approximately 7–9 million km^2^, wetlands are extremely important to human life, covering approximately 45% of the natural value of ecosystems [8,9].

Despite their important roles, wetlands are home to the some of the most vulnerable ecosystems on Earth [10]. Approximately 50% of wetlands have been lost since the 1900s [11], and the rate of area decline and degradation occurs faster than in any other ecosystem [12,13]. The degradation, destruction, and transformation of wetlands are driven by anthropogenic and natural factors, which are directly related to changes in temperature, transpiration, and precipitation [[14], [15], [16]]. Human impacts not only directly affect wetland ecosystems through industrial discharge activities [17] but also indirectly through changes to climate, for example rainfall patterns [18]. In South America, the growing population and demand for agricultural products have driven infrastructure development, farmland expansion, and natural resource exploitation, placing wetlands under immense pressure. Climate change and extreme weather events, such as droughts and storms, also lead to changes in the area and quality of wetlands or ecosystems in river basins [19]. The destruction and degradation of wetlands reduce their ability to provide ecosystem services, including declining flood and drought regulation services, services that provide biodiversity, and reduced natural resource supplies. Even the Pantanal, the world's largest wetland, has suffered from the harsh effects of droughts and wildfires, threatening some species that are on the brink of extinction [20]. A study conducted in China's Lixiahe wetlands revealed that the vegetation area was reduced by 69%, mainly due to land use changes for aquaculture development [21].

Owing to the important role of wetland ecosystems, especially mangroves, in providing economic and environmental value, mangrove restoration projects have been implemented in several countries. Mangrove restoration projects after the 2004 Indonesian tsunami [22] or Super Typhoon Haiyan in the Philippines in 2013, which mainly involved growing *Rhizophora stylosa* [23], were deployed to restore areas of wetland ecosystems that were lost to these natural disasters. In Mexico, mangrove restoration has been undertaken recently, mostly with financial support from federal agencies, for carbon management [24]. However, mangrove restoration projects have not always been particularly effective because of a lack of understanding of the reasons for mangrove loss, poor species and site selection, and a lack of incentive for local people to participate in the long-term management of restored areas [25].

With advances in remote sensing and geographic information systems (GIS), satellite imagery has been applied to the study of the status quo and fluctuations in forest or wetland areas in several regions and countries [[26], [27], [28], [29], [30]]. Currently, there are more than 300 Earth observation satellites from more than 15 countries active [31], data from some of which are available commercially, while others are free. Traditional methods for monitoring and mapping are often time-consuming, labour-intensive, and expensive, with limited spatial ranges, implying that changes are not detected across large areas [32]. Song et al. applied SPOT 6 imaging to analyse chlorophyll-a levels in Kristalbad, an artificial wetland between Hengelo and Enschede in the Netherlands [33]. Similarly, satellite imagery from, for example, FORMOSAT-2, SPOT 6, Sentinel-2MSI, and LANDSAT-8 has been used to monitor water bodies in addition to traditional analyses using simple hydrological models [[34], [35], [36], [37], [38], [39]].

Mangroves are an important type of wetland in Vietnam. This high-value ecosystem provides many important ecosystem services, such as carbon storage, wood supply, and habitat for many marine species, thus aiding in the maintenance of coastal stability and control of coastal erosion [40,41]. However, the mangrove area has declined sharply over time, from 408,500 ha (1943) to 290,000 ha (1962), 252,000 ha (1982), and 155,290 ha (2000). During the 60-year period from 1943 to 2003, Vietnam's mangroves decreased by 4/5 (IUCN, 2012). Shrimp farming [42] and projects for the development of industrial and urban parks were the main causes of the decline, especially from 1985 to the present (IUCN, 2012). The area covered by natural mangroves in Vietnam is still declining. According to statistics, only 21% of the existing mangrove areas in Vietnam are natural forests, while the rest are replanted [43].

One of the richest and most important mangrove habitats in northern Vietnam is found in the Dong Rui commune, which is in the Tien Yen district of Quang Ninh Province. However, from 1992 until now, many policies for economic development and land use conversion have greatly affected the area and quality of the wetland ecosystem [44]. To this day, how specific areas have changed or how much people have helped or hurt the wetland ecosystem in the study area have not been evaluated. Using satellite images to keep track of how different land use types change over time can help us to determine how policies affect wetland ecosystems.

Thus, the objectives of this study were to assess wetland ecosystem fluctuations using remote sensing imagery and identify the causes of fluctuations in wetland ecosystems in the Dong Rui commune during 1975–2022. We believe that the use of generally available remote sensing data to study anthropogenic impacts on land cover and land use provides a novel and cost-effective tool for applying similar techniques elsewhere. Several studies in Vietnam have only assessed the variability in wetland ecosystems relying on remote sensing images without explaining what caused the changes. Thus, our results provide a basis for the developing sustainable development strategies for the conservation of wetland ecosystems in the Quang Ninh Province.

## 2 Data and methods

### 2.1 Study area

**Fig. 1:**
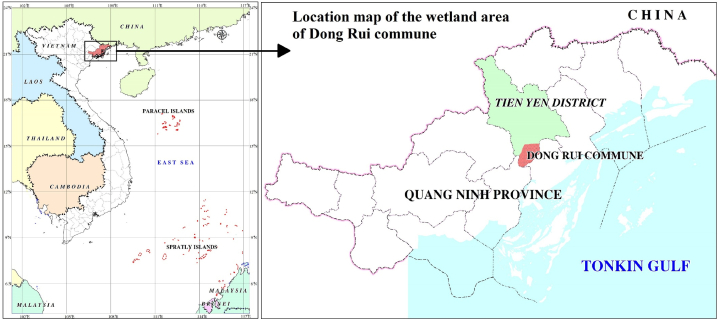
Map of the Dong Rui commune wetland area.

The Dong Rui commune wetland covers an area of 4,902 ha, with geographical coordinates from 21°11′N to 21°33′N and from 107°13′E to 107°32′E [45] (Fig. 1). This is a coastal accretion area, with low terrain, gradually moving to the sea, with an average elevation of 1.5–3 m. Many places have been converted into arable land and aquaculture lagoons, and the rest are parrot beaches and coastal dunes flooded by tidal water. The salty soil group dominates the research area, while the alluvial soil in the central area is where the local population was concentrated. The Dong Rui region is characterised by a tropical monsoon climate, with hot and humid summers and dry and cold winters. The average annual temperature is approximately 23–24 °C. The highest temperatures are observed in July and August (average 28–29 °C), while the lowest are in January and December (average 15–16 °C). The winters are cold and foggy, and the average January temperature ranges from 14 to 18 °C.

The wetland of the Dong Rui area is affected by two river basins: the Tien Yen River and the Ba Be River. In terms of the dry season (exhaust season), the low water level, limited flow, and saltwater intrusion due to the tide are the greatest, creating favourable conditions for brackish water aquaculture. In contrast, in the rainy season, frequent flood events occur, which do not last because the flood is fast and recedes quickly; however, the narrow riverbed can cause flooding in some places, adversely affecting the aquatic environment [45].

In estuary biomes, abundant mangroves appear in tidal wetlands, where they can adapt to unstable environmental conditions, and are affected by incoming and outgoing tides. Due to the terrain conditions of narrow estuary marshes distributed along the riverbank, the vegetation pattern tends to be a group of trees that grow scattered and take a form of strips. The biome of the area includes characteristics plant species, including *Aegiceras corniculatum, Kandelia obovata*, *Bruguiera gymnornitreza*, and *Rhizophora stylosa*.” [44].

### 2.2 Data

**Fig. 2:**
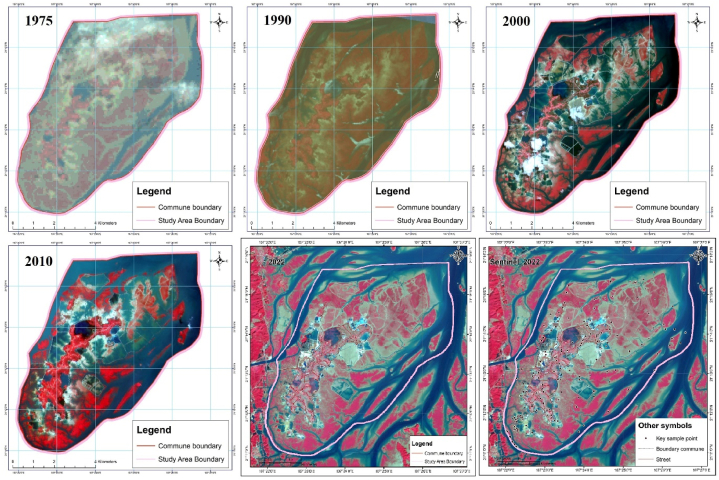
Free multitemporal remote sensing photos from 1975 to 2022 in the Dong Rui commune wetland area.

In this study, we used the satellite imaging data types LANDSAT_LM2 in 1975, SPOT-2 in 1990, SPOT-4 in 2000 and 2010, and Planet in 2022 to map the cover of the Dong Rui commune wetland ecosystem (Fig. 2 and Table 1).

**Table 1:** Specifications of remote sensing images.

Date	Sensor	Scene ID	Image bands	Resolution
April 20, 1975	LANDSAT_LM2	LM21350451975110AAA05	B4, Green (500–600 nm) B5, Red (600–700 nm) B6, Near IR (700–800 nm)	All bands: 60 m × 60 m
July 08, 1990	SPOT-2	22723089008070340292 P	Pan (510–730 nm) Green (500–590 nm) Red (610–680 nm) Near IR (780–890 nm)	Panchromatic: 10 m × 10 m Multispectral: 20 m × 20 m
January 14, 2000	SPOT-4	42723080001140324441I	Pan (610–680 nm) Green (500–590 nm) Red (610–680 nm) Near IR (780–890 nm) SWIR (1,530–1,750 nm)	Panchromatic: 10 m × 10 m Multispectral: 20 m × 20 m
August 30, 2010	SPOT-4	42733081008300310592I	Blue (0.455–0.525 μm) Green (0.530–0.590 μm) Red (0.625–0.695 μm) Near-Infrared (0.760–0.890 μm)	Panchromatic: 10 m × 10 m Multispectral: 20 m × 20 m
April 06, 2022	Sentinel-2	20221221T032141	Blue (0.455–0.525 μm) Green (0.530–0.590 μm) Red (0.625–0.695 μm) Near-Infrared (0.760–0.890 μm)	Panchromatic: 10 m × 10 m Multispectral: 20 m × 20 m

### 2.3 Mapping of wetland ecosystem cover

Jalbuena et al. used a multiresolution algorithm to segment LiDAR images based on recognition software [46]. In the present study, eCognition Developer v 9.1 software was used to segment and interpret photos to map the status of wetland cover in the Dong Rui commune in 1975, 1990, 2000, 2010, and 2022 based on multitemporal satellite imagery.

The image-interpreting key template (MKA) used for 2022 photo analysis was included in the Collect Earth software. We assigned a status to 80% of the total number of templates after fragmentation. The remaining 20% of MKA (184 samples) was used to verify the scene and assess accuracy. For the 1975, 1990, 2000, and 2010 MKA sets, each year's MKA sets were included in Collect Earth software, which interprets the MKA status quo. From 2000, 2010, and 2022, Google photos was the primary source for the MKA status quo, with the MKA set for the 1990 reference using SPOT and LANDSAT photos to assign status. MKA for 1975 referencing used LANDSAT images to assign the status on a year-by-year basis.

Based on the map of the current state of land use in the Dong Rui commune in 2022, a ratio of 1/10.000 (established by the Tien Yen District People's Committee), the compilation of the same circle of the same state was implemented, and photo fragmentation was performed for years to limit errors in the photo interpretation process. We assigned a status to the MKA set for each year and initiated the process of interpreting the image.

### 2.4 Random forest algorithm

Random forest is an algorithm that combines tree prediction factors such that each tree depends on the values of a random vector that is sampled independently and has the same distribution for all trees in the forest. The generalisation error of the tree classifiers depends on the predominance of each tree in the forest and the correlation between them [47]. The general principle of random forest is the synthesis of a set of random decision trees. As individual trees are randomly disturbed, the forest benefits from a broader exploration of the space of all possible tree predictors, delivering better predictive performance [48].

### 2.5 Verification of classification results

Congalton (1999) asserts that constructing a matrix of confusion between the classification outcome, the Kappa coefficient (K) test, and the assessment sample is the best technique for judging accuracy [49]. To test the results of image interpretation, the random selection method was used; each state had at least 10 points, and the status quo in the field was verified and compared with the results of the disbursement. If the accuracy was less than 75%, then it was necessary to review the sampling process and the method of obtaining the decompression key to improve the accuracy of the post-classification map [50]. This process required the following steps:

- Overall Accuracy
- User Accuracy
- Producer Accuracy

The Kappa coefficient was used as a measure of the classification accuracy. This is the utility coefficient of all elements from the error matrix, as well as the fundamental difference between what is real about the deviation error of the matrix and the total number of changes indicated by the rows and columns [51]. The Kappa coefficient is defined as follows:

(1) $$K=\frac{N\sum_{i=1}^{r}X_{ii}-\sum_{i=1}^{r}\left(X_{i+}-X_{+i}\right)}{N^{2}-\sum_{i=1}^{r}\left(X_{i+}-X_{+i}\right)}\text{,}$$

where r is the number of columns in the image matrix; X_ii_ is the number of pixels observed in row i and column i (on the main diagonal); X_i+_ is the total number of pixels observed in row i; X is the total number of pixels observed in column i; and N is the total number of pixels observed in the image matrix.

The K index is typically between 0 and 1; the obtained value was in this range, and the accuracy of the classification was acceptable. According to the U.S. Geological Survey, K has three value groups: K ≥ 0.8, high accuracy; 0.4 ≤ K < 0.8, moderate accuracy; and K < 0.4, low accuracy.

### 2.6 Field data collection

**Fig. 3:**
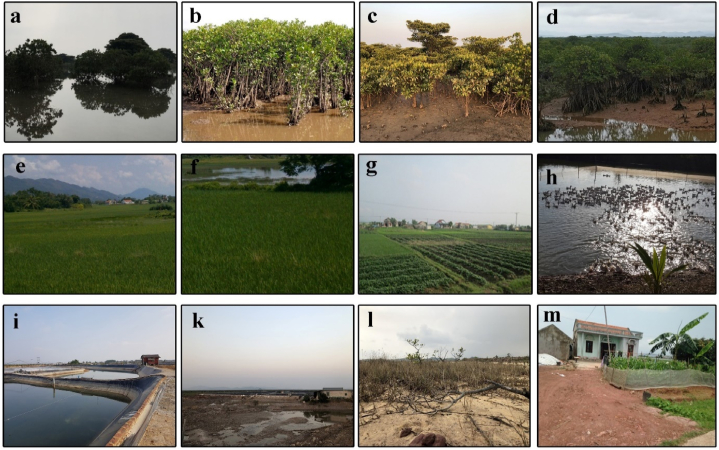
Actual images verifying the photo decoding key template set in Dong Rui. a, b, c, d: Form of locking to interpret mangrove photos; e, f, g: Agricultural land photo decoding key; h, i, k: Sample of the lock to interpret the photograph of aquaculture lagoon; l: Other land image interpretation key; m: Sample of the land photo decoding key in rural land.

To reverse the classification results of the photo interpretation course sample set (184 samples), a field trip was conducted in May and December 2022 to the Dong Rui commune. Based on the actual test results, a classification test was conducted, and the error matrix of the image interpretation process was calculated (Fig. 3). In addition to field sampling for map accuracy assessment, documents and data on mangrove restoration projects in the Dong Rui Commune since 1996 were collected.

## 3 Result

### 3.1 Status and fluctuation of the wetland ecosystem of the Dong Rui commune during 1975–2022

#### 3.1.1 Image classification and disassembly inspection

Based on the results of the image analysis and formulas for calculating coefficient K (Formula 3), an overall accuracy index matrix table was developed to map the current state of the Dong Rui wetland ecosystem cover described in 2022 (Table 2). Table 2 shows that the overall accuracy value in 2022 was 92.3%, corresponding to K = 0.91. Mangrove objects are easily recognizable, with a total of 39/40 samples interpreting the photos correctly. Other land types had a higher rate of confusion; when interpreting agricultural land, rural land, or plantation forests, 3/20 samples were misinterpreted. Similar to agricultural land, 2/20 samples were misinterpreted as rural land or mangrove. The group of aquaculture lagoons and water surfaces was highly accurate, with only 1/25 of the interpretation samples being incorrect. There was one sample where an aquaculture lagoon was identified as a water surface and one sample of a water surface interpreted as an aquaculture lagoon.

**Table 2:** Error matrix describing wetland ecosystem cover in 2022.

		Ground Reference								Total
		Others land	Newly plantation forest	Water surface	Agricultural land	Aquaculture lagoon	Rural land	Mangrove	Plantation forest	
Classification	Others land	**17**	0	0	1	0	0	0	0	**18**
	Newly plantation forest	0	**16**	0	0	0	0	1	1	**18**
	Water surface	0	0	**24**	0	1	0	0	0	**25**
	Agricultural land	1	0	0	**18**	0	1	0	0	**20**
	Aquaculture lagoon	0	0	1	0	**24**	0	0	0	**25**
	Rural land	1	0	0	1	0	**15**	0	0	**17**
	Mangrove	0	0	0	1	0	0	**39**	1	**41**
	Plantation forest	1	1	0	0	0	0	0	**18**	**20**
**Total**		20	17	25	21	25	16	40	20	**184**
User Accuracy (%)		85.00	94.12	96.00	85.71	96.00	93.75	97.50	90.00	**92.26**
Producer Accuracy (%)		94.44	88.89	96.00	90.00	96.00	88.24	95.12	90.00	**92.34**
**Overall Accuracy (%)**		**92.30**								

Bolded values emphasize the accuracy of the image verification process.

For the 1990, 2000, and 2010 maps, comparative data were used from the 1990s, 2000, and 2010 land use cover maps provided by the Dong Rui Commune People's Committee. For 1975 alone, comparative data were used from 1:10,000 scale topographic maps established in 1975. The overall accuracy for 1975, 1990, 2000, and 2010 was 87.65, 90.32, 91.03, and 91.16, respectively; the Kappa coefficient was 0.85, 0.88, 0.90, and 0.90, respectively.

#### 3.1.2 Map of the current state of Dong Rui wetland ecosystem cover

**Fig. 4:**
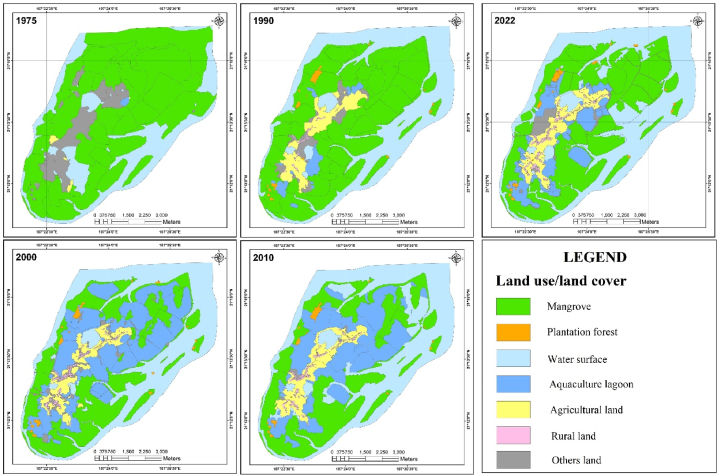
Maps of Dong Rui commune wetland ecosystem cover from 1975 to 2022.

Based on the process of interpreting multitemporal remote sensing images and evaluating the results of image classification, a map of the mantle of the Dong Rui wetland ecosystem was produced for 5 years: 1975, 1990, 2000, 2010, and 2022 (Fig. 4).

**Fig. 5:**
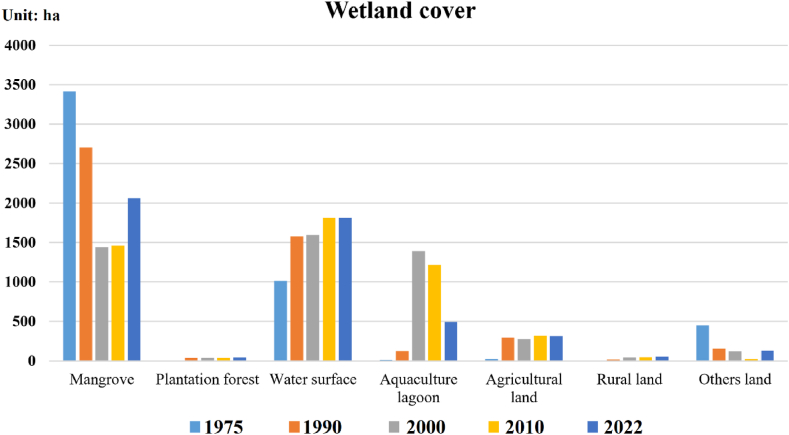
Status and fluctuation of the wetland ecosystem cover in the Dong Rui commune from 1975 to 2022.

According to Fig. 4, Fig. 5, the types of wetland ecosystem cover in the study area changed significantly between 1975 and 2022. In particular, the area of mangrove forests and aquaculture lagoons showed a strong change at different stages due to the Dong Rui commune land-use policies for each period.

The area of land type in rural areas also tended to increase gradually with each stage, and the area of agricultural land also tended to increase gradually. The area covered by plantation forests (mainly *Acacia auriculiformis*) began to appear around the 1990s in low-hilly areas in the Dong Rui commune area, tending to maintain the overall stability of the area. The water surface area also tended to increase gradually, and the total area increased sharply between 1975, 1990, and 2000–2010. Other types of land (including vacant land and unused land) had the largest area in 1975 and began to trend downwards until 2010.

#### 3.1.3 Wetland ecosystem cover fluctuations during 1975–2022

Based on the maps of the state of wetland ecosystem cover over 5 years (1975, 1990, 2000, 2000, 2010, and 2022), fluctuations were observed in the Dong Rui wetland ecosystem cover over four periods: 1975–1990, 1990–2000, 2000–2010, and 2010–2022 (Table 3, Table 4, Table 5, Table 6).

**Table 3:** Fluctuation of the wetland ecosystem cover in the Dong Rui commune during 1975–1990 (unit: ha).

Year	In 1990								
In 1975	**Wetland ecosystem cover**	Mangrove	Plantation forest	Water surface	Aquaculture lagoon	Agricultural land	Rural land	Others land	**Total**
	Mangrove	2,552.36	8.59	716.29	61.84	33.63	1.15	38.83	**3,412.69**
	Plantation forest	0.00	0.00	0.00	0.00	0.00	0.00	0.00	**0.00**
	Water surface	101.40	0.64	853.97	37.64	3.78	0.00	13.26	**1,010.69**
	Aquaculture lagoon	10.13	0.00	0.00	0.00	0.57	0.00		**10.70**
	Agricultural land	4.18	1.01	0.06	0.08	2.82	0.12	11.52	**19.79**
	Rural land	0.00	0.00	0.00	0.00	0.00	0.00	0.00	**0.00**
	Others land	33.61	26.82	3.05	25.80	253.50	14.68	90.67	**448.13**
	**Total**	**2,701.68**	**37.06**	**1,573.37**	**125.36**	**294.30**	**15.95**	**154.28**	**4,902.00**

(Values show the area of converted land use from 1975 to 1990).

**Table 4:** Fluctuation of the wetland ecosystem cover in the Dong Rui commune during 1990–2000 (unit: ha).

Year	In 2000								
In 1990	**Wetland ecosystem cover**	Mangrove	Plantation forest	Water surface	Aquaculture lagoon	Agricultural land	Rural land	Others land	**Total**
	Mangrove	1,389.37	3.20	73.11	1,182.00	12.42	0.00	41.58	**2,701.68**
	Plantation forest	0.50	29.67	0.11	6.54	0.06	0.00	0.18	**37.06**
	Water surface	27.58	3.03	1,518.63	13.52	6.42	0.00	4.19	**1,573.37**
	Aquaculture lagoon	23.83	1.26	1.78	85.74	7.46	0.00	5.29	**125.36**
	Agricultural land	0.05	0.00	0.43	49.61	181.10	19.03	44.08	**294.30**
	Rural land	0.00	0.00	0.00	0.00	0.00	15.95	0.00	**15.95**
	Others land	0.16		0.26	54.00	68.47	7.79	23.60	**154.28**
	**Total**	**1,441.49**	**37.16**	**1,594.32**	**1,391.41**	**275.93**	**42.77**	**118.92**	**4,902.00**

(Values show the area of converted land use from 1990 to 2000).

**Table 5:** Fluctuations of the wetland ecosystem cover in the Dong Rui commune during 2000–2010 (unit: ha).

Year	In 2010								
In 2000	**Wetland ecosystem cover**	Mangrove	Plantation forest	Water surface	Aquaculture lagoon	Agricultural land	Rural land	Others land	**Total**
	Mangrove	1,309.23	0.52	53.76	77.81	0.10	0.00	0.07	**1,441.49**
	Plantation forest	2.80	29.38	2.75	2.23	0.00	0.00	0.00	**37.16**
	Water surface	40.57	0.04	1,543.05	9.84	0.78	0.00	0.04	**1,594.32**
	Aquaculture lagoon	83.65	5.62	198.62	1,050.82	43.80	0.00	8.90	**1,391.41**
	Agricultural land	4.42	0.00	10.31	35.43	219.94	1.96	3.87	**275.93**
	Rural land	0.00	0.00	0.00	0.00	0.00	42.77	0.00	**42.77**
	Others land	15.88	0.37	5.07	38.45	51.30	0.00	7.85	**118.92**
	**Total**	**1,456.55**	**35.93**	**1,813.56**	**1,214.58**	**315.92**	**44.73**	**20.73**	**4,902.00**

(Values show the area of converted land use from 1990 to 2000).

**Table 6:** Fluctuation of the wetland ecosystem cover in the Dong Rui commune during 2010–2022 (unit: ha).

Year	In 2022								
In 2010	**Wetland ecosystem cover**	Mangrove	Plantation forest	Water surface	Aquaculture lagoon	Agricultural land	Rural land	Others land	**Total**
	Mangrove	1,318.37	4.69	85.43	39.21	0.43	0.00	8.42	**1,456.55**
	Plantation forest	0.59	31.73	1.23	1.43	0.03	0.00	0.92	**35.93**
	Water surface	222.50	2.15	1,572.42	11.71	2.53	0.00	2.25	**1,813.56**
	Aquaculture lagoon	519.59	1.73	144.25	411.11	39.50	0.66	97.74	**1,214.58**
	Agricultural land	0.07	0.20	8.63	26.78	258.41	9.17	12.66	**315.92**
	Rural land	0.00	0.00	0.00	0.00	0.00	44.73	0.00	**44.73**
	Others land	0.06		0.69	2.42	12.11	0.00	5.45	**20.73**
	**Total**	**2,061.18**	**40.50**	**1,812.65**	**492.66**	**313.01**	**54.56**	**127.44**	**4,902.00**

(Values show the area of converted land use from 1990 to 2000).

From Table 3, during the 15-year period (1975–1990), the area of mangrove forest in the study area lost 711 ha, which was mainly exploited, leading to a conversion to water surface area. In addition, some areas have been converted to land-making purposes in rural areas, as well as to aquaculture lagoons. Other types of land were also converted to many different types, the largest of which was to agricultural land (253.50 ha). A total of 101.4 ha of water surface was converted to mangroves, mainly on the estuary side. Aquaculture lagoons began to appear more commonly, mainly converted from mangrove areas, water surfaces, and other land. Rural land began to appear during this period (15.95 ha), which was largely converted from other land (14.68 ha). This was the period of establishment of the Dong Rui commune (in 1978), and people began to arrive and settle, concentrated mainly in the centre of the commune.

From 1990 to 2000, there was marked conversion of all types of cover in the study area. As shown in Table 4, approximately 1,182 ha of mangroves was converted to aquaculture lagoons. In addition, 73.11 ha of mangrove forests was exploited or lost from natural forces and converted to water surfaces. The area of agricultural land was also transformed into many other types of land use, including conversion to aquaculture lagoons (49.61 ha) and rural land. Other types of land were also converted into aquaculture lagoons (54 ha). The area of land in rural areas increased significantly and nearly tripled between 1990 and 2000 (15.95 ha in 1990 to 42.77 ha in 2000).

Between 2000 and 2010, the area of mangrove forests in the study area began to recover, with nearly 130 ha converted from different types of land use to mangroves (Table 5). However, there were still some areas of mangrove forests that continued to be converted to other uses, such as aquaculture lagoons (77.81 ha), as well as those exploited or lost due to natural events and converted to water surfaces (53.76 ha) or to other land types (16.7 ha). The area of other land types was reduced nearly six-fold, and was mainly converted to agricultural land, aquaculture lagoons, or mangrove plantations. The plantation forest area (mainly *Acacia auriculiformis* and *Acacia mangium*) tended to remain stable, providing a source of income for local households. The area of aquaculture lagoons began to decrease (by approximately 180 ha) and was largely abandoned, becoming water surface areas, planted mangrove forests, or agricultural land. In general, during this period, the area of aquaculture lagoons tended to decrease, and the area of mangrove forests began to increase, although not significantly.

During 2010–2022, the mangrove area was significantly restored, with a total of 604.63 ha of mangroves recovered (Table 3, Table 6). Abandoned aquaculture lagoons and water surface areas converted into mangrove forests accounted for the majority (519.59 ha and 225.5 ha, respectively). The area of aquaculture lagoons was reduced by more than 720 ha, mainly converted to mangrove forests, simply abandoned, or switched to other types of land use. Land use types such as farming or rural land remained relatively stable. Plantations tended to decrease because of a lack of economic efficiency.

### 3.2 Impact of developmental policies and programs on the study area during 1975–2022

In 1978, the Dong Rui commune was established in the Tien Yen district, Quang Ninh Province, Vietnam. By 1992, when identifying aquaculture as a key economic sector, the Dong Rui Commune People's Committee granted 1,500 ha of mangrove forest area to households in the commune and investment lagoon owners, which were converted into aquaculture lagoons, especially for shrimp farming.

In 1996 and 1997, the Dutch Science and Technology Commission (KWT) funded the commune with two plantings of 40 ha of forest in Ha village, Village Four, where the distance between the trees and the rows was 1.3 × 1.3 m (Table 7). By 1998, the KWT had funded the planting of 50 ha in Village Four. The Mangrove Action and Restoration Organization, Japan funded two reforestation sessions, the first planting of more than 40 ha of forest, where the distance between the trees was 0.7 × 0.7 m, and the second planting of 60 ha of mangrove forest in the central pine and upper villages. The area of salty plantations had not expanded further; rather, it had shrunk because of the clearing of natural mangrove forests and the destruction of plantation forests for aquaculture lagoon development.

**Table 7:** Statistics on the area of mangrove planting stages in the Dong Rui commune.

№	Year	Sponsoring Agency	Area (ha)
1	1996	Dutch Science and Technology Commission - KWT of the Netherlands	40
2	1997	–	7
3	1998	Dutch Science and Technology Commission - KWT of the Netherlands	60
4	1998	ACMANG	40
5	1998	ACMANG	60
6	2005	UNDP	50
7	2007	UNDP	30
8	2007	ACMANG	45
9	2010	UNDP	20
10	2014	ACMANG	100
11	2015	–	2
12	2017	–	5
13	2018	–	31.4
14	2020	–	30
**Total**			**452**

Since 2000, the Dong Rui commune government has made policy adjustments, calling for a number of investment projects by the government and non-governmental organisations to restore the destroyed mangrove areas. By 2005, the KWT, ACMANG, the Center for Environmental Resource Research (Hanoi National University), the Vietnam Institute of Forestry Sciences, and the UNDP Development Program had implemented and supported the cultivation and restoration of local mangrove areas (Table 7).

As early as 2006, The People's Committee of Dong Rui Commune issued Decision No. 368-QD/UB dated May 10, 2006 ‘On the allocation of land and mangrove forests to the village community’, the commitment to protect the forests of the commune. This is an important policy for the protection and development of community-based mangroves.

Additionally, in 2006, the Asia-Pacific Mangrove Ecosystem Integrated Research and Training Program (UNDP/UNESCO) agreed to fund the commune to plant 50 ha of *Kandelia obovata* forest in a central village, which was implemented in early 2007. In the same year, 45 ha of *R. stylosa* forest, funded by the ACMANG, was deployed in the study area (Table 7).

In 2010, the UNDP funded the planting of 20 ha of *R. stylosa* and *K. obovata* forests in Ha and Trung villages with improved planting and care techniques that have resulted in high efficiency. In 2014, the Dong Rui commune continued to plant 100 ha of forest with capital funded by ACMANG. Since then, in combination with funding organisations, local authorities have organised many plantations to restore the mangrove forest area in the whole commune, with a total area of 68.4 ha (Table 7).

To recover the area of aquatic lagoons and expand the area of mangrove forests, by 2012 the Dong Rui commune recovered more than 1,000 ha. Simultaneously, propagation, raising awareness, encouraging people to replant forests, and releasing shrimp and fish under the mangrove forest canopy in a natural way are promoted, both to protect forests and to develop the economy from forests. Consequently, the land area for mangrove forest development in the study area has increased to nearly 2,700 ha, which is 55% of the natural land area of the commune.

The project to plant and restore coastal mangrove ecosystems in Quang Ninh Province was supported by the Mangrove Ecosystem Research Department (MERD/CRES) and the Mangrove Action and Restoration Organization (ACMANG), Japan, in terms of funding and engineering, implemented in Quang Ninh from 1999 to the present. Currently, the project has planted more than 1000 ha of mangrove forests in the coastal communes of Quang Ninh Province, including part of the Dong Rui commune.

## 4 Discussion

Wetland ecosystems in several parts of the world are highly degraded, in that, they cannot be restored naturally [52]. Determining the extent and causes of fluctuations in wetland ecosystems is the basis for identifying solutions for restoring these highly sensitive ecosystems. Multitemporal remote sensing imaging is a suitable tool for monitoring and assessing fluctuations in wetlands [[53], [54], [55]]. Research using such methods can provide a basis for the management, supervision, and conservation of wetland ecosystems by managers and local authorities. Based on multitemporal remote sensing documents, a map of the current state of wetland ecosystem cover was developed for the Dong Rui commune. Together with the random forest algorithm, building land use land cover maps have been built for 1975, 1990, 2000, 2010, and 2022 with a coefficient of K ranging from 0.85 to 0.91 (the highest is in 2022, K = 0.91). Combining plant cover classification and algorithms helps improve the accuracy of the current land use land cover map [48].

Based on maps of the wetland cover of the Dong Rui commune from 1975 to 2022, we not only identified fluctuation trends but also found the causes of the fluctuations in land cover for each stage. From 1975 to 1990, the land use structure changed little, mainly due to a small population and sparse settlement, and the impact on wetland resources was limited because the commune was only established in 1978. The main period of mangrove forest loss was from 1990 to 2000, with nearly 1,200 ha of mangrove area removed. In 1992, the local government implemented a policy to convert mangrove forests into aquaculture lagoons. This was the main cause of the severe deterioration of the mangrove forest area in the locality. This finding is also in line with the conclusions of many other studies that reported aquaculture, especially shrimp farming, to be one of the leading causes of the decline in wetland area and quality [21,42]. In addition, the conversion from wetlands to agricultural land in rural and urban areas is a common cause of decline in wetland ecosystems worldwide [56]. The continuous exploitation of natural resources, waste from industrial activities, and unsustainable economic activities are the main causes of increased sensitivity and risk to coastal ecosystems [17,[57], [58], [59], [60], [61]].

Since 2000, the mangrove forest area was gradually restored, reducing the area of aquaculture lagoons as a result of reforestation policies and the attraction of investment capital to support the restoration of mangrove forest areas, with many abandoned aquaculture lagoons being converted to mangrove forests due to a lack of economic efficiency. The policy of allocating land and mangrove forests to the village community brings benefits, raising people's awareness and making the protection of mangroves a community responsibility [44]. More than 500 ha of mangrove forests was restored as a result of this policy [45]. Artificial mangrove plantations are the most effective way to restore natural forests [62]. Many countries have implemented reforestation projects, including Sri Lanka [63], Guyana, Brazil (North Shelf) [64], and Mexico (Pantanos de Centla Biosphere Reserve in Tabasco) [65].

Policies for sustainable livelihoods, such as aquaculture below the forest canopy as well as using mangrove forests to develop community ecotourism, are increasingly effective for protecting mangrove forests. In recent years, Quang Ninh Province has also focused on developing wetland ecosystems in the study area, with plans to establish a wetland area in the Dong Rui commune adjacent to the second RAMSAR area of northern Vietnam [44]. The establishment of nature reserves with supporting policy tools is an effective measure for protecting and restoring wetland ecosystems [66], and sanctuaries have been established to protect and restore lost wetland ecosystems in several countries, such as in Indonesia [67], New Zealand [68], and China [69]. An effective ecosystem management plan should also aim to include means to limit the damage people cause to aquatic ecosystems [70]. Assessment of different human activities is the basis for determining the causes of environmental pollution and degradation for wetland ecosystems globally [71,72]. This is considered the most important step in the protection and development of wetland ecosystems, with increasing diversity being considered the second most important aspect in northern Vietnam.

The integration of multitemporal remote sensing methods with field verification surveys allows us to assess the status quo and fluctuations in wetland ecosystems during specific periods and is the basis for forecasting future trends under the influence of natural factors and anthropogenic activities, particularly the impact of development policies. This provides a basis for planning strategies to conserve the biodiversity of wetland ecosystems, contributing to sustainable development associated with community livelihoods. However, the use of satellite imagery also has certain limitations, such as medium resolution, which is suitable only for large-scale areas. For small-scale areas, unmanned aerial vehicle imagery can be applied to accurately determine area fluctuations, crop health, or the development of additional mangrove plantations.

## 5 Conclusion

Establishing a map of the status quo and assessing wetland cover fluctuations are the basis for the management and conservation of biodiversity and sustainable development of coastal areas. In this study, we assessed the changes in the wetland ecosystem in Dong Rui commune from 1975 to 2022 based on freely available satellite images. Accordingly, we evaluated and explained the causes of changes in wetland areas due to different anthropogenic impacts. We argue here that using medium-resolution satellite imagery to monitor annual fluctuations in wetland ecosystems is highly efficient and cost-effective.

Policies to protect, restore, and develop mangrove forests have affected the conservation of wetland ecosystems. Afforestation and mangrove protection projects have contributed to the restoration of mangrove areas, creating the premise for restoring wetland ecosystems and improving environmental quality. Establishing protected areas is also an effective solution for the conservation and restoration of wetland ecosystems.

Monitoring the development of additional mangrove plantation areas allows us to determine how effective wetland ecosystem restoration projects have been. However, in this study, the data from satellite images could not help to determine the survival rate of mangrove trees in the areas of mangrove restoration. In the future, the use of drones will be an effective tool for monitoring the growth and development of mangrove plantations on a small scale.

## Author contribution statement

Dung Trung Ngo; Hoi Dang Nguyen: Conceived and designed the experiments;

Dung Trung Ngo; Hoi Dang Nguyen: Performed the experiments;

Dung Trung Ngo; Huan Cao Nguyen: Analyzed and interpreted the data;

Dung Trung Ngo; Hoi Dang Nguyen: Contributed reagents, materials, analysis tools or data; Dung Trung Ngo; Huan Cao Nguyen; Hoi Dang Nguyen: Wrote the paper

## Data availability statement

Data included in article/supp. material/referenced in article.

## Declaration of competing interest

The authors declare that they have no known competing financial interests or personal relationships that could have appeared to influence the work reported in this paper.
